# Anandamide modulation of monocyte-derived Langerhans cells: implications for immune homeostasis and skin inflammation

**DOI:** 10.3389/fimmu.2024.1423776

**Published:** 2024-06-24

**Authors:** Zsófia Pénzes, Dorottya Horváth, Petra Molnár, Tünde Fekete, Kitti Pázmándi, Attila Bácsi, Attila Gábor Szöllősi

**Affiliations:** ^1^ Department of Immunology, Faculty of Medicine, University of Debrecen, Debrecen, Hungary; ^2^ Doctoral School of Molecular Medicine, University of Debrecen, Debrecen, Hungary; ^3^ ELKH-DE Allergology Research Group, Debrecen, Hungary

**Keywords:** anandamide, Langerhans, cells, CD1a, epidermis, skin immunology

## Abstract

**Introduction:**

The endocannabinoid system (ECS), named after the chemical compounds found in the cannabis plant, is a regulatory network of neurotransmitters, receptors, and enzymes that plays crucial roles in skin health and disease. Endogenous ligands of the ECS, called endocannabinoids, have proven to be important regulators of immune responses. One of the most prevalent endocannabinoids, arachidonoylethanolamide (also known as anandamide), is known for its anti-inflammatory effects. Langerhans cells (LCs) are the sole antigen-presenting cells present in the human epidermis. They serve as the first line of defense against pathogens and are essential for the skin’s specific immune responses and play a critical role in maintaining tissue homeostasis; however, little is known about the effect of endocannabinoids on these cells. Our research aimed to provide the connection between monocyte-derived Langerhans cells (moLCs) and the ECS, shedding light on their collaborative roles in immune homeostasis and inflammation.

**Methods:**

Human monocytes were differentiated into moLCs using established protocols. Anandamide was applied during the differentiation process to test its effect on the viability, marker expression, and cytokine production of the cells, as well as in short term treatments for intracellular calcium measurement. TLR ligands applied after the differentiation protocol were used to activate moLCs. The impact of anandamide on the functionality of moLCs was further assessed using differential gene expression analysis of bulk RNA-Seq data, moLC-T cell cocultures, while ELISpot was employed to determine polarization of T cells activated in the aforementioned cocultures.

**Results:**

Anandamide did not significantly affect the viability of moLCs up to 10 µM. When applied during the differentiation process it had only a negligible effect on CD207 expression, the prototypic marker of LCs; however, there was an observed reduction in CD1a expression by moLCs. Anandamide had no significant effects on the maturation status of moLCs, nor did it affect the maturation induced by TLR3 and TLR7/8 agonists. MoLCs differentiated in the presence of anandamide did however show decreased production of CXCL8, IL-6, IL-10 and IL-12 cytokines induced by TLR3 and TLR7/8 activation. Anandamide-treated moLCs showed an increased capability to activate naïve T cells; however, not to the level seen with combined TLR agonism. RNA sequencing analysis of moLCs differentiated with anandamide showed modest changes compared to control cells but did reveal an inhibitory effect on oxidative phosphorylation specifically in activated moLCs. Anandamide also promoted the polarization of naïve T cells towards a Th1 phenotype.

**Discussion:**

Our results show that anandamide has nuanced effects on the differentiation, maturation, cytokine secretion, metabolism and function of activated moLCs. Among these changes the decrease in CD1a expression on moLCs holds promise to selectively dampen inflammation induced by CD1a restricted T cells, which have been implicated as drivers of inflammation in common inflammatory skin conditions such as psoriasis, atopic dermatitis and contact dermatitis.

## 1 Introduction

The endocannabinoid system (ECS) is a remarkably complex regulatory network of neurotransmitters, receptors, and enzymes that plays an important role in maintaining homeostasis within the human body. Through its various elements the ECS modulates a wide range of physiological processes, contributing to overall health and well-being. The discovery of the ECS revolutionized our understanding of how cannabinoids, both external and internal, interact with the human body. This regulatory system influences a wide range of functions, including pain modulation, appetite, mood regulation, sleep, memory, the immune response and inflammation (1, 2).

The ECS takes its name from cannabinoids, the chemical compounds found in the cannabis plant, which interact with the system’s receptors. The ECS is not solely influenced by external cannabinoids, it also produces endogenous ligands, known as endocannabinoids (ECs). These ECs, such as N-arachidonoylethanolamine (anandamide, AEA) and 2-arachidonoylglycerol (2-AG), are synthesized on-demand in response to specific physiological signals (3). The ECs mainly act on two cannabinoid receptors, the CB1 and CB2 receptors, which were discovered in the late 20th century (4–6).

Most ECs are produced from membrane precursors in response to stimuli, although anandamide can be stored in the cell (7). The concentration of ECs in the body fluctuate greatly, and is influenced by many factors. As a consequence serum anandamide levels generally range from 1 to 5 nM, and 2-AG levels from 10 to 500 nM (8).

The ECS has significant impact on skin homeostasis. As our body’s largest organ, skin serves as a protective barrier between our internal and the external environment. Recent studies have revealed an intriguing connection between the skin and the ECS (9, 10). Beyond its role in physical defense, the skin is a dynamic and complex organ involved in numerous physiological processes, including temperature regulation, sensation, and the orchestration of immune responses. The presence of the ECS in the skin signifies its importance in maintaining the intricate balance of skin functions. Both CB1 and CB2 receptors, the primary receptors of the ECS, have been identified in various components of the skin, including epidermal keratinocytes, sebaceous glands, hair follicles, and sensory nerve fibers. The ECS is involved in the regulation of skin cell proliferation, survival and differentiation (11). This widespread distribution suggests that the ECS plays a significant role in regulating multiple aspects of skin physiology. Fine-tuning endocannabinoid tone appears to be a key factor in modulating skin growth and differentiation (12, 13).

One key area where the ECS exerts its influence is in the regulation of inflammation in the skin. Inflammatory skin conditions, such as acne, eczema, psoriasis, and allergic dermatitis, involve an immune response gone awry. Studies have shown that activation of cannabinoid receptors in the skin can help modulate immune responses, reducing excessive inflammation. This suggests that targeting the ECS may hold promise in developing novel anti-inflammatory treatments for skin disorders (9).

Local regulation of immune cells by endocannabinoid mediators is a well-documented phenomenon. The most common ECs (anandamide and 2-AG) are characterized by a dominant anti-inflammatory effect, as they reduce the production of pro-inflammatory cytokines in many immune cells, induce cell migration or even cell apoptosis (14). Anandamide at low, nanomolar concentrations (3–30 nM) reduced the production of interleukin (IL-6) and IL-8, but at higher concentrations (0.3–3 μM) inhibited the production of tumor necrosis factor alpha (TNFα), interferon γ (IFNγ), IL-4 and soluble p75 TNFα receptors (15). Anandamide has also been shown to suppress the release of IL-2, TNFα and IFNγ cytokines from activated human peripheral T lymphocytes, mainly through a CB2-dependent mechanism (16). It also effectively suppressed the *in vitro* induction of IL-6 and the release of nitric oxide (NO) from macrophages triggered by lipopolysaccharides (17). Primary human monocytes show increased production of NO via the CB1 receptor upon administration of increasing doses of 2-AG. Interestingly, these cells become rounded and immobile upon 2-AG treatment, which appears to be immunosuppressive in terms of decreased cytokine production and adhesion (18). 2-AG also strongly suppresses Toll-like receptor (TLR)9-mediated type I IFN production by human primary plasmacytoid dendritic cells. The effect is mediated exclusively by CB2, and the abolition of this 2-AG inhibition has been shown to be key in the pathogenesis of systemic lupus erythematosus (SLE) (19).

Although its effects on cell function may vary and even be controversial in some cases, it is generally agreed that the ECS acts as a kind of endogenous brake on both innate and adaptive immune processes, as detailed above.

LCs are professional antigen presenting cells derived from yolk sac precursors similarly to tissue resident macrophages, but functionally they share many characteristics with dendritic cells (DCs). They can be identified primarily based on their expression of CD1a and langerin/CD207 in the stratum spinosum of the epidermis and in the mucosa, where their main function is antigen presentation to T cells after their activation (20). The efficient antigen presentation of LCs to CD4^+^ and CD8^+^ T cells highlights their important role in the induction of helper T cell (Th) mediated regulatory and humoral immune responses, as well as in the induction and maintenance of cytotoxic T cell immunity (21–23). LCs that cross-present antigens are also essential for the initial reactivation of resident CD8^+^ memory T cells in the epidermis (24). LCs can migrate to surrounding lymph nodes mediated by CCR7 to activate naïve T cells by antigen presentation (25). More recent research has proposed that there are two steady-state LC subtypes in the epidermis of human skin (CD1a^high^langerin^high^ LC1 and CD1a^high^langerin^int^ LC2), with higher CD1c expression and better Birbeck granule organization in LC1 vs. LC2 (26).

LC-like cells can also be derived from myeloid precursors. Specifically, CD14^+^ monocytes from adult peripheral blood undergo differentiation into monocyte-derived LCs (moLCs) when exposed to granulocyte/macrophage colony-stimulating factor (GM-CSF), IL-4, and transforming growth factor β (TGF- β) (27–29). There is no standard protocol widely adopted among research groups for generating moLCs (27, 30–33). Our research group worked to refine protocols to ensure the accuracy and reproducibility of the moLC experiments (34). In alignment with the existing literature, our study demonstrates a striking resemblance between the model cells we employed and the previously mentioned LC2 subtype.

As detailed above both LCs and the ECS are fascinating components of the human body’s (immune) system. Nonetheless, the effect of ECs (i.e. anandamide and 2-AG) has not yet been investigated in LCs, the only professional antigen-presenting cell in the skin (35–37).

Understanding the interaction between the ECS and LCs holds promise to unveil a fascinating avenue to explore to help maintain skin health, and potentially to develop innovative therapeutic interventions targeting LCs. Furthermore, understanding the ECS’s intricate mechanisms provides a foundation for exploring the therapeutic potential of ECs in treating various inflammatory skin diseases.

## 2 Materials and methods

### 2.1 Reagents and antibodies

Anandamide was purchased from ChemFaces (Wuhan, Hubei, PRC), whilst 2-AG was purchased from Cayman Chemical (Ann Arbor, MI). The ECs were diluted in absolute ethanol. The final concentration of absolute ethanol in the culture medium was 1:1000.

Fetal Bovine Serum (FBS, Gibco™), and cell culture media (RPMI1640) were purchased from ThermoFisher Scientific (Waltham, MA). IL-6, IL-10, IL-12, CXCL8 ELISA kits were all from BD-Biosciences (San Jose, CA, USA). Polyinosinic:polycytidylic acid (p(I:C)) (TL3 agonist) and CL075 (TLR7/8 agonist) were obtained from Invivogen (San Diego, CA), which were dissolve in nuclease-free water.

Fluorescently labeled monoclonal antibodies (mAbs) against CD83 (RRID: AB_314514), CD1a (RRID: AB_314020), CD207 (RRID: AB_2561590) were sourced from BioLegend (San Diego, CA, USA), HLA-DQ (RRID: AB_2573320) mAbs were obtained from ThermoFisher Scientific, while CD86 (RRID: AB_2275742) and CCR7 (RRID: AB_2259847) was from R&D Systems (Minneapolis, MI, USA).

### 2.2 Isolation of monocytes and differentiation of moLCs

Heparinized leukocyte-enriched buffy coats were obtained from consenting healthy individuals. The process received approval from the Regional Blood Center of the Hungarian National Blood Transfusion Service (Debrecen, Hungary), sanctioned by both the Head of the National Transfusion Service and the Regional and Institutional Ethics Committee of the University of Debrecen’s Faculty of Medicine (Debrecen, Hungary; approval number: OVSZK 3572–2/2015/5200).

Peripheral blood monomorphonuclear cells (PBMC) were isolated from buffy coats through Ficoll gradient centrifugation. Monocytes were then derived from PBMCs using immunomagnetic beads linked to anti-CD14 antibodies (Miltenyi Biotec, Bergisch Gladbach, Germany), following the manufacturer’s instructions.

Primary human moLCs were differentiated from monocytes following established protocols (34). Briefly, monocytes were cultured in 12-well plates with RPMI 1640 medium (Sigma-Aldrich, St. Louis, MI) containing 10% heat-inactivated FBS, 10 mM HEPES, 100 μg/ml penicillin/streptomycin, 50 mM 2-mercaptoethanol (all from Sigma-Aldrich), GM-CSF (80 ng/ml) (Gentaur Molecular Products, London, UK), and TGF-β1 (10 ng/mL), TNFα (20 ng/mL), IL-4 (20 ng/ml for 48 hrs) (PeproTech, Brussels, Belgium) at a density of 1 × 10^6^ cells/ml for 5 days. On day 2, half of the culture medium was replaced with fresh medium containing the full cytokine amount. As a positive control of maturation, p(I:C) (20 µg/ml) and CL075 (0.5 µg/ml) were applied both for 24 hours on day 4.

The impact of anandamide on the differentiation process was evaluated by adding anandamide (10 µM) or the vehicle (absolute ethanol) alone on days 0, 2, and 4 of the protocol, and determining the expression of langerin/CD207. Maturation markers (CCR7, CD83, CD86, HLA-DQ) were assessed on day 5 using fluorescence-activated cell sorting (FACS) with a 2000R Novocyte Flow cytometer (ACEA Biosciences, San Diego, CA).

### 2.3 Analysis of cell viability

Cell viability was assessed by 7-aminoactinomycin D (7-AAD; Sigma–Aldrich). Cells were cultured in 12-well monocytes were cultured in 12-well plates in RPMI 1640 medium (Sigma-Aldrich, St. Louis, MI) containing 10% heat-inactivated FBS, 10 mM HEPES, 100 μg/ml penicillin/streptomycin, 50 mM 2-mercaptoethanol (all from Sigma-Aldrich), GM-CSF (80 ng/ml) (Gentaur Molecular Products, London, UK) and TGF-β1 (10 ng/mL), TNFα (20 ng/mL), IL-4 (20 ng/ml for 48 hrs) (PeproTech, Brussels, Belgium) at a density of 1 × 10^6^ cells/ml for 5 days. MoLCs were treated with 10 µM concentration of anandamide over the course of their differentiation. The cells were stained in 50 µL of PBS based buffer for 15 min at room temperature in the dark with 10 µg 7-AAD. Fluorescence was detected using a Novocyte 2000R flow cytometer (ACEA Biosciences). Data were analyzed with FlowJo 10.8.1 software (FlowJo).

Cell viability was evaluated using PrestoBlue Cell Viability Reagent (ThermoFisher, excitation at 560 nm and emission at 590 nm). PrestoBlue® is a ready-to-use, cell-permeable, resazurin-based solution that acts as a cell viability indicator by utilizing the reducing power of living cells to quantitatively measure cell proliferation. Cells were seeded in quadruplicate in 96-well black-well/clear-bottom plates on day 5 (Greiner Bio-One, Kremsmünster, Austria). The assay was performed according to the manufacturer’s protocols. Fluorescence measurements were taken using an EnVision 2105 Multimode Plate Reader (Perkin Elmer, Waltham, MA, USA).

### 2.4 Isolation of naïve CD4^+^ T cells and moLC-T cell coculture

Naïve CD4+ T cells were isolated for the mixed leucocyte reaction (MLR) through negative selection from PBMC using a human Naïve CD4+ Isolation Kit (Miltenyi Biotec), following the manufacturer’s protocol.

In the proliferation assay, naïve T cells were labelled with 0.5 µM carboxyfluorescein succinimidyl ester (CFSE, ThermoFisher Scientific). MoLCs were cultured with naïve T cells at a 1:6 ratio of moLCs to naïve T cells in 96-well round-bottom cell culture plates with 200 μl RPMI 1640 medium for 5 days. The co-cultures were supplemented with 1 μg/ml anti-human CD3 mAb (BD Biosciences, Franklin, Lakes, NJ, United States). On day 5, T cells were collected, and fluorescence intensities were measured on the FL1 (530 ± 15 nm) channel using a Novocyte 2000R flow cytometer. Data were analyzed with FlowJo 10.8.1 software (FlowJo, Ashland, OR, USA).

### 2.5 Enzyme-linked ImmunoSpot assay

To determine T cell polarization, naïve CD4+ T cells were seeded in 96-well round-bottom cell culture plates with moLCs at a 1:6 ratio in 200 μl RPMI 1640 medium for 3 days. After 3 days of co-culturing, the cells underwent washing and were then reseeded on an ELISpot plate coated with 0.5 μg/ml mouse anti-human CD3 antibody and incubated for 24 hours. Analysis of Th1 responses was conducted using an avidin-horseradish peroxidase-based enzyme-linked ELISpot system, and the results were read with an ImmunoScan plate reader (Cell Technology Limited, Hong Kong, Kowloon, SAR).

### 2.6 Intracellular Ca^2+^ measurement

Monocytes were cultured in the presence of GM-CSF, TNFα, TGFβ and IL-4 (for 48 hrs) for 5 days to generate moLCs. Cells were seeded in Ibidi chamber (µ-Slide VI 0.) (Ibidi, Gräfelfing, Germany) at a density of 5 × 10^4^ cells/ml for 3 hrs at 37°C and stained with Fluo-4 (ThermoFisher Scientific) Ca^2+^ ion indicator. Time lapse measurement of intracellular Ca^2+^ were assessed by measuring the fluorescence intensity using an Olympus IX-81 Microscope. Images were captured every second for a total measurement time of 5 minutes. Anandamide and TLR agonists were applied around the 1 minute mark after baseline fluorescence was established.

### 2.7 Flow cytometry analysis

Staining took place in a phosphate-buffered saline (PBS) buffer enriched with 2% (v/v) heat-inactivated FBS and 2 mM EDTA (pH 7.4). Cells were subjected to staining on ice for 20 minutes, followed by two washes with a PBS-based buffer. The cells were then resuspended in 100 μl of the buffer and maintained on ice until measurement.

Measurements were conducted using a Novocyte 2000R flow cytometer from ACEA Biosciences. The obtained results were analyzed using FlowJo 10.8.1 software.

### 2.8 Enzyme-linked immunosorbent assay

Cytokine levels from moLCs were assessed using ELISA, with supernatants collected on day 5 of the experiment. ELISA kits for IL-6, IL-10, IL-12 and CXCL8 were sourced from BD Biosciences, and measurements were carried out in accordance with the manufacturer’s instructions. The EnVision 2105 Multimode Plate Reader (Perkin Elmer) was utilized for quantifying cytokine levels, and concentrations were calculated using the cubic logistic model (38) with GraphPad Prism 9.1.2 for Windows (GraphPad Software).

### 2.9 Analysis of signal transduction pathways based on differential gene expression- RNA sequencing

The moLC samples for RNA-Seq analysis were collected on day 5. Total RNA isolation was performed by conventional chloroform-isopropanol fraction separation followed by RNA integrity verification (Eukaryotic Total RNA Nano Kit (Agilent Technologies, Waldbronn, Germany) with Agilent BioAnalyzer. RNA-Seq libraries were performed using the Illumina NextSeq 500 sequencing platform. For sequencing data quality and further statistical analysis, StrandNGS software (www.strand-ngs.com) was used. As a first step, differential gene expression analysis was performed using limma-voom package. In this step lowly expressed genes were filtered based on count-per-million reads, with the filtering threshold set to 1.0 in a minimum of two samples. Adjusted p values were calculated based on Benjamini and Hochberg method (39), and the threshold for significant change was set at 0.05 or below. Library preparation, sequencing and primary data analysis were performed at the Genomic Medicine and Bioinformatics Services Laboratory, University of Debrecen.

Primary component analysis was performed using the pcaExplorer package, using default settings (40). Volcano plots were generated with the help of the VolcaNoseR web app (41), genes with -log_10_ *P*>2 and fold change>2, or with fold change<-2, were highlighted as significantly changed. Gene set enrichment analysis was performed using the ShinyGO web app (41), with an FDR cutoff of 0.05, a minimum and maximum pathway size of 2 and 5000, respectively. Pathway database was used for gene counts for GO analysis.

### 2.10 Statistical analysis

The statistical evaluation in the mentioned study was conducted using GraphPad Prism 9.1.2 for Windows (GraphPad Software Inc., La Jolla, CA, USA). The study utilized one-way ANOVA to compare two groups and assess groups of three or more. *Post-hoc* analyses were performed using either Tukey’s or Dunnett’s test. Statistical significance was determined at a significance level of P < 0.05, unless otherwise indicated.

## 3 Results

### 3.1 Anandamide decreases CD1a expression, increases maturation markers on moLCs without affecting their viability

**Figure 1:**
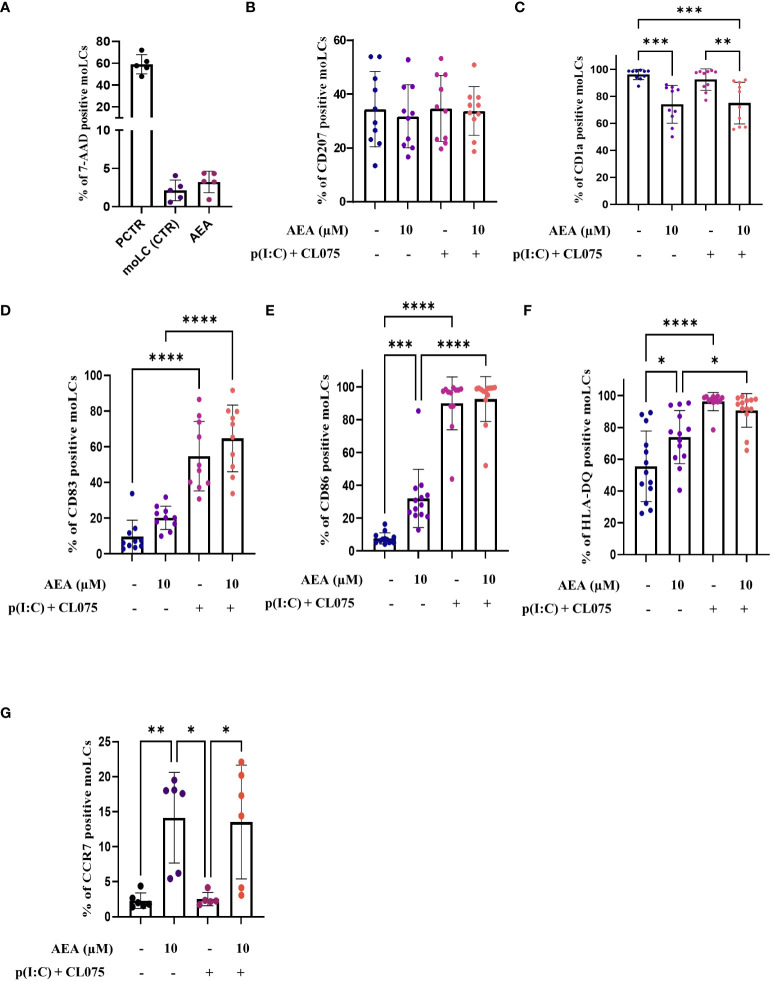
Anandamide decreases CD1a expression, increases maturation markers on moLCs without affecting their viability. Monocytes were cultured in the presence of GM-CSF, TNFα, TGFβ and IL-4 (for 48 hrs) for 5 days to generate moLCs in the presence of 10 µM AEA, vehicle (0.1 v/v% absolute ethanol). Maturation was induced on day 4 by p(I:C) (20 µg/ml) and CL075 (0.5 µg/ml), both applied for 24 hours. N≥10 mean ± SD. Percentage of cells positive for 7-AAD **(A)**, CD207 **(B)**, CD1a **(C)**, CD83 **(D)**, CD86 **(E)**, HLA-DQ **(F)**, CCR7 **(G)** following the indicated treatments. *p < 0.05, ** p < 0.01, ***p < 0.001, **** p<0.0001 as indicated (determined by repeated measures one-way ANOVA). Individual donors are represented by symbols. 7-AAD, 7-aminoactinomycin; PCTR, Positive control; cells damaged by freezing (30 min), AEA, N-arachidonoylethanolamine, anandamide; CL075, TLR7/8 agonist; p(I:C), Polyinosinic:polycytidylic acid, TLR3 agonist.

Based on literature data, we know that both anandamide and 2-AG have complex effects on biological processes in immune cells (see above). We investigated the effect of both anandamide and 2-AG applied to moLCs during their differentiation process in several experiments over a wide concentration spectrum, i.e. from 0.1 μM to 10 μM (**Figure 1**, **Supplementary Figure 1**). Since no significant effect was observed on either cell differentiation or maturation with 2-AG (**Supplementary Figures 1A-E**), as compared to anandamide (**Supplementary Figures 1F-J**) 2-AG treatment was excluded from further experiments.

To rule out the possibility that the observed effects were due to changes in cell viability, we also performed 7-AAD staining on control, and moLCs differentiated in the presence of the highest dose of anandamide used in the above experiments (i.e. 10 μM), which showed that anandamide does not influence cellular viability (**Figure 1A**; **Supplementary Figure 2**).

Subsequently, we delved into the impact of anandamide on the differentiation and maturation processes of moLCs, first by investigating the expression of characteristic differentiation markers CD207 and CD1a (**Figures 1B, C**). When anandamide was used at high concentrations throughout the differentiation of the cells, we found that it had only a negligible effect on CD207 (**Figure 1B**); however, it was associated with reduced CD1a expression by moLCs (**Figure 1C**). To determine the maturation status of the cells we investigated the expression of the activation marker CD83, the costimulatory molecule CD86, and the MHC class II receptor HLA-DQ (**Figures 1D-F**) at the end of the differentiation process. As anandamide alone did not induce the maturation of the cells, to assess its putative anti-inflammatory activity TLR3 and TLR7/8 agonists (p(I:C) and CL075, respectively) were applied to differentiated moLCs to induce maturation. This combination of TLR agonists was effective at inducing the maturation of moLCs, as the expression of the CD83, CD86, and HLA-DQ were all significantly increased by their application (**Figures 1D-F**). Unsurprisingly, activation of differentiated moLCs with TLR agonists did not counteract the effect of high doses of anandamide applied throughout their differentiation on the expression of CD207 and CD1a (**Figures 1B, C**), as the decrease in CD1a levels was still apparent, concomitant with negligible effects on CD207 expression. MoLCs differentiated in the presence of anandamide did not show reduced responsiveness to TLR activation, as none of the tested markers showed decreased expression compared to control moLCs activated with TLR agonists (**Figures 1D-F**).

We extended these assessments to CCR7, a chemokine receptor that facilitates the exodus of LCs from the skin to draining lymph nodes. Intriguingly, we found that anandamide greatly and significantly increased the expression of CCR7 when applied during differentiation, which effect persisted after activation with TLR agonists (TLR3+ TLR7/8; **Figure 1G**). We also performed RNA-Seq evaluation of moLCs treated as described above (detailed below), and from these data we found that the cells express multiple other chemokine receptors. Specifically moLC express *CCR1*, *CCR2*, *CCR3*, *CCR4*, *CCR5*, *CCR6*, C*CR7, CXCR1, CXCR2, CXCR3, CXCR4, CXCR5, ACKR3, ACKR4, CX3CR1, XCR1*, and *GPR35/CXCR8*. No expression was found for *CCR8, CCR9, CCR10, CXCR6, ACKR1, ACKR2*. In control cells the highest expression based on raw read counts can be determined for *CCR1*, *CCR5*, *CXCR4*, *CCR7* and *GPR35/CXCR8*. Of the expressed receptors several showed increased expression after treatment with p(I:C) and CL075 (*CCR4, CCR6, CCR7, CXCR4, AKCR3, AKCR4*), while others were downregulated by the activation of the cells (*CCR1*, *CCR2*, *CCR5*, *CXCR1*, *CXCR2*, *CXCR5*), and some showed no significant change among the four groups (*CCR3*, *CXCR3*, *CX3CR1, GPR35/CXCR8*). Heatmap of normalized gene expression calculated by the limma-voom package is presented in **Supplementary Figure 3**.

Interestingly, the mRNA expression of *CCR7* showed minimal elevation in the moLCs differentiated in the presence of anandamide, but was increased in cells treated with TLR agonists. In moLCs differentiated in the presence of anandamide and treated with TLR agonists the expression of *CCR7* is similar to the level measured in TLR agonist treated cells. This discrepancy between the protein and mRNA levels is likely because both samples were collected at the same time, in other words anandamide possibly caused an increase in *CCR7* expression before the measured time point.

### 3.2 AEA can decrease CXCL8, IL-6, IL-10 and IL-12 cytokine production induced by TLR3 and TLR7/8 activation on moLCs

**Figure 2:**
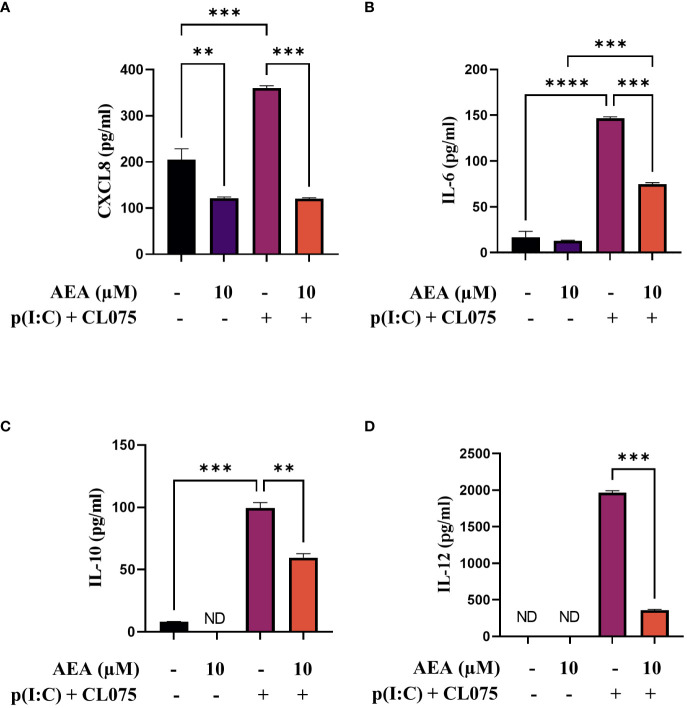
AEA can decrease CXCL8, IL-6, IL-10 and IL-12 cytokine production induced by TLR3 and TLR7/8 activation on moLCs. Monocytes were cultured in the presence of GM-CSF, TNFα, TGFβ and IL-4 (for 48 hrs) for 5 days to generate moLCs in the presence of 10 µM AEA, vehicle (0.1 v/v% absolute ethanol). Maturation was induced on day 4 by p(I:C) (20 µg/ml) and CL075 (0.5 µg/ml), both applied for 24 hours. N≥3 mean ± SD. CXCL8 **(A)**, IL-6 **(B)**, IL-10 **(C)** and IL-12 **(D)** production was determined with ELISA from supernatants. Bar plots represent mean ± SD of representative results from N≥3 independent experiments, ** p < 0.01, ***p < 0.001, **** p<0.0001 compared to the marked groups as determined by repeated measures one-way ANOVA. ND, not determined. AEA, N-arachidonoylethanolamine, anandamide; CL075: TLR7/8 agonist, p(I:C), Polyinosinic:polycytidylic acid, TLR3 agonist.

Antigen presenting cells, such as moLCs, are known to produce chemokines and cytokines that can influence the migration and activation of inflammatory cells, as well as polarization of naïve T cells activated by the presented antigens. Investigating the chemokine/cytokine production of our moLCs we found that cells differentiated in the presence of anandamide showed decreased baseline CXCL8 production, without influencing the production of IL-6, IL-10 or IL-12 (**Figures 2A-D**, first two bars). Interestingly, although combined TLR agonist treatment significantly increased the secretion of all investigated cytokines in control cells, anandamide had a general inhibitory effect, as these cells showed decreased secretion of all aforementioned mediators (**Figures 2A-D**, third and fourth bars).

### 3.3 Anandamide causes delayed calcium transients in moLCs

**Figure 3:**
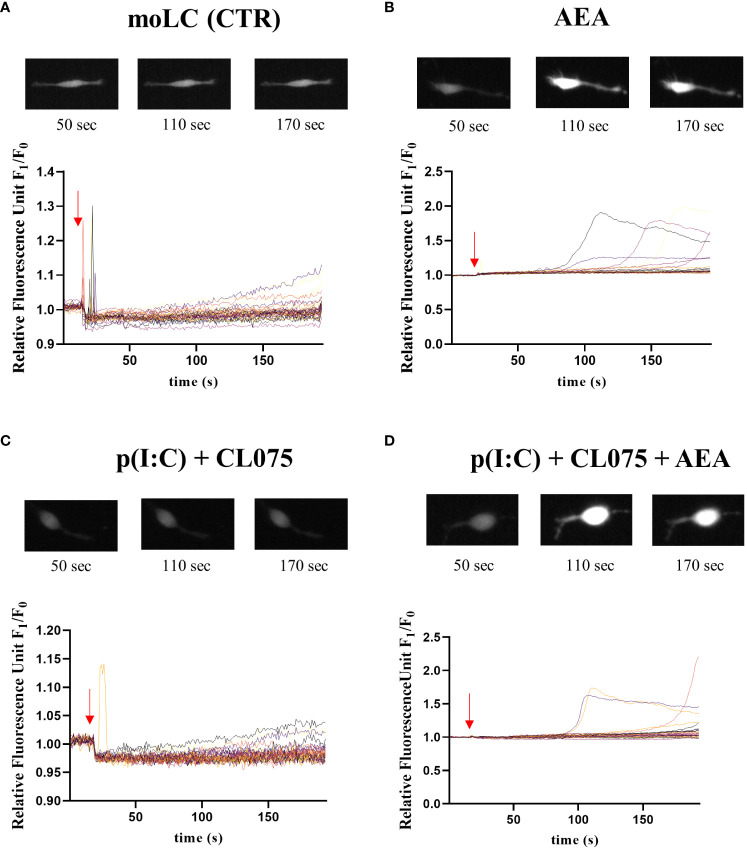
Anandamide causes delayed calcium transients in moLCs. Monocytes were cultured in the presence of GM-CSF, TNFα, TGFβ and IL-4 (for 48 hrs) for 5 days to generate moLCs. Cells were seeded at a density of 5 × 10^4^ cells/ml in Ibidi μ-Slide chambers on day 5. Representative graphs showing changes in intracellular Ca^2+^ concentration in response to vehicle (0.1 v/v% absolute ethanol) **(A)**, 10 µM AEA **(B)**, p(I:C) (20 µg/ml) and CL075 (0.5 µg/ml) **(C)** and the three compounds together (AEA + p(I:C) + CL075) **(D)** as determined by measuring changes in Fluo-4 fluorescence in real time by Olympus IX-81 Microscope. Arrows show the approximate time of application of treating compounds. Representative images above the line graphs show typical changes in fluorescence intensity at the marked time points. AEA, N-arachidonoylethanolamine, anandamide; CL075: TLR7/8 agonist, p(I:C), Polyinosinic:polycytidylic acid, TLR3 agonist.

Anandamide has been shown to interfere with calcium signaling in cells, which could be one of the reasons behind the general decrease in cytokine production. One of the possible targets of anandamide is the non-specific cation channel transient receptor potential vanilloid 1 (TRPV1), which is mostly permeable to calcium. The application of anandamide to moLCs did not induce a quick increase in intracellular calcium, although some cells showed a delayed signal. This is more in line with a downstream effect, rather than a direct opening of an ion channel (**Figure 3**, **Supplementary Videos 1**-**4**).

### 3.4 Anandamide treatment enhances T cell proliferation-inducing capability of moLCs

**Figure 4:**
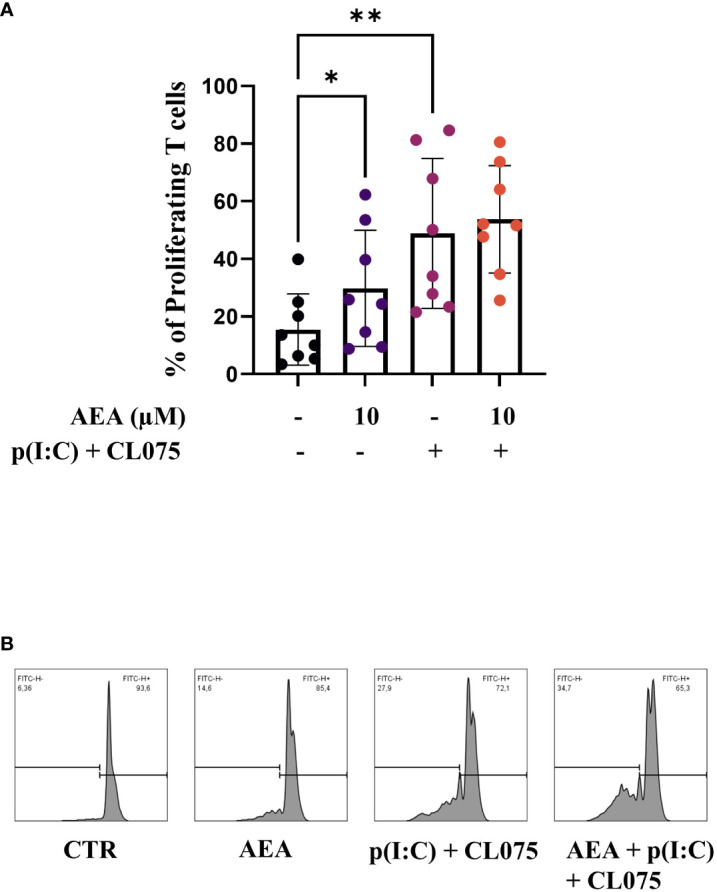
Anandamide treatment enhances T cell proliferation-inducing capability of moLCs. Monocytes were cultured in the presence of GM-CSF, TNFα, TGFβ and IL-4 (for 48 hrs) for 5 days to generate moLCs in the presence of 10 µM AEA, vehicle (0.1 v/v% absolute ethanol). Maturation was induced on day 4 by p(I:C) (20 µg/ml) and CL075 (0.5 µg/ml), both applied for 24 hours. Percentage of proliferating T cells after 5 days of coculture of naïve T cells and moLCs at a ratio of 6:1 **(A)**. Representative histograms after 5 days co-culture of naïve T cells and moLCs at a ratio of 6:1 **(B)**. N=8, mean ± SD, individual donors are represented by symbols. * p<0.05, ** p < 0.01 as determined by repeated measures one-way ANOVA compared to the marked groups. AEA, N-arachidonoylethanolamine, anandamide; CL075: TLR7/8 agonist, p(I:C), Polyinosinic:polycytidylic acid, TLR3 agonist.

As LCs are professional antigen presenting cells, we investigated naïve T cell proliferation induced by moLCs using a CFSE proliferation assay. MoLCs differentiated in the presence of anandamide induced more naïve T cell proliferation compared to the control, although not to the level seen after combined TLR agonism. TLR agonists did not show a significantly stronger effect on anandamide-treated moLCs, although we did observe a minimal increase between the two groups (**Figure 4A**, representative histogram plots are shown on **Figure 4B**).

### 3.5 Anandamide treatment alone results in modest changes to the transcriptome, but inhibits oxidative phosphorylation in activated moLCs as determined by RNASeq

From the findings presented above, it appears that anandamide applied during the differentiation process induces somewhat conflicting effects. On the one hand, it enhances antigen presentation and consequently T cell proliferation, aspects that may be viewed as fostering inflammatory responses. Conversely, cytokine production appears to diminish with anandamide treatment. To unravel and understand the underlying mechanisms, we undertook RNA sequencing.

**Figure 5:**
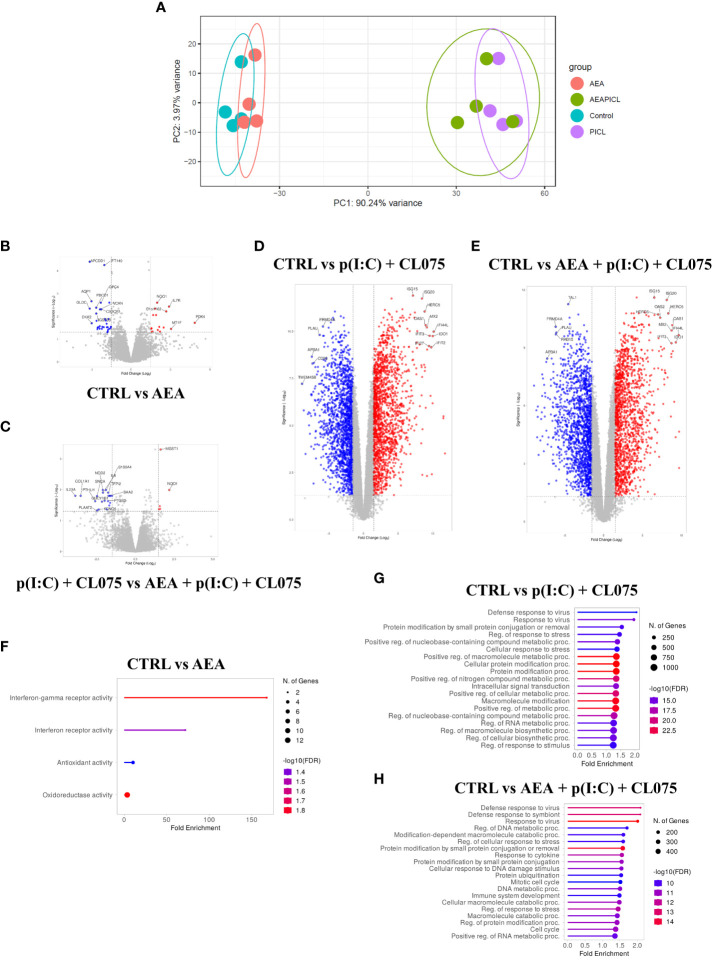
Anandamide treatment alone results in modest changes to transcriptome as determined by RNASeq. Monocytes were cultured in the presence of GM-CSF, TNFα, TGFβ and IL-4 (for 48 hrs) for 5 days to generate moLCs in the presence of 10 µM AEA, vehicle (0.1 v/v% absolute ethanol). Maturation was induced on day 4 by p(I:C) (20 µg/ml) and CL075 (0.5 µg/ml), both applied for 24 hours. The gene expression of vehicle-treated (blue) and AEA-treated moLCs (red) cluster together in PCA, as does that of mature moLCs with, or without AEA co-treatment (green and purple, respectively) **(A)**. A confidence ellipse is drawn for each group with a confidence interval level of 0.95. Volcano plots of RNASeq data with the top 15 most highly changed genes highlighted for each plot. Red dots indicate genes with log_10_ *P*>2 and fold change>2, while blue dots indicate genes with -log_10_ *P*>2 and fold change<-2, when compared to vehicle-treated control cells **(B-D)**, or to matured cells that did not receive AEA treatment **(E)**. Lollipop graphs of upregulated pathways as determined by Gene Ontology analysis in AEA treated cells compared to vehicle-treated control **(F)**, p(I:C) and CL075, and p(I:C), CL075 and AEA treated cells compared to vehicle-treated control (**G** and **H**, respectively). The size of the circle corresponds to the number of induced genes, while the color of the line corresponds to the -log10 of the False Discovery Rate (FDR). AEA, N-arachidonoylethanolamine, anandamide; CL075: TLR7/8 agonist, p(I:C), Polyinosinic:polycytidylic acid, TLR3 agonist.

**Figure 6:**
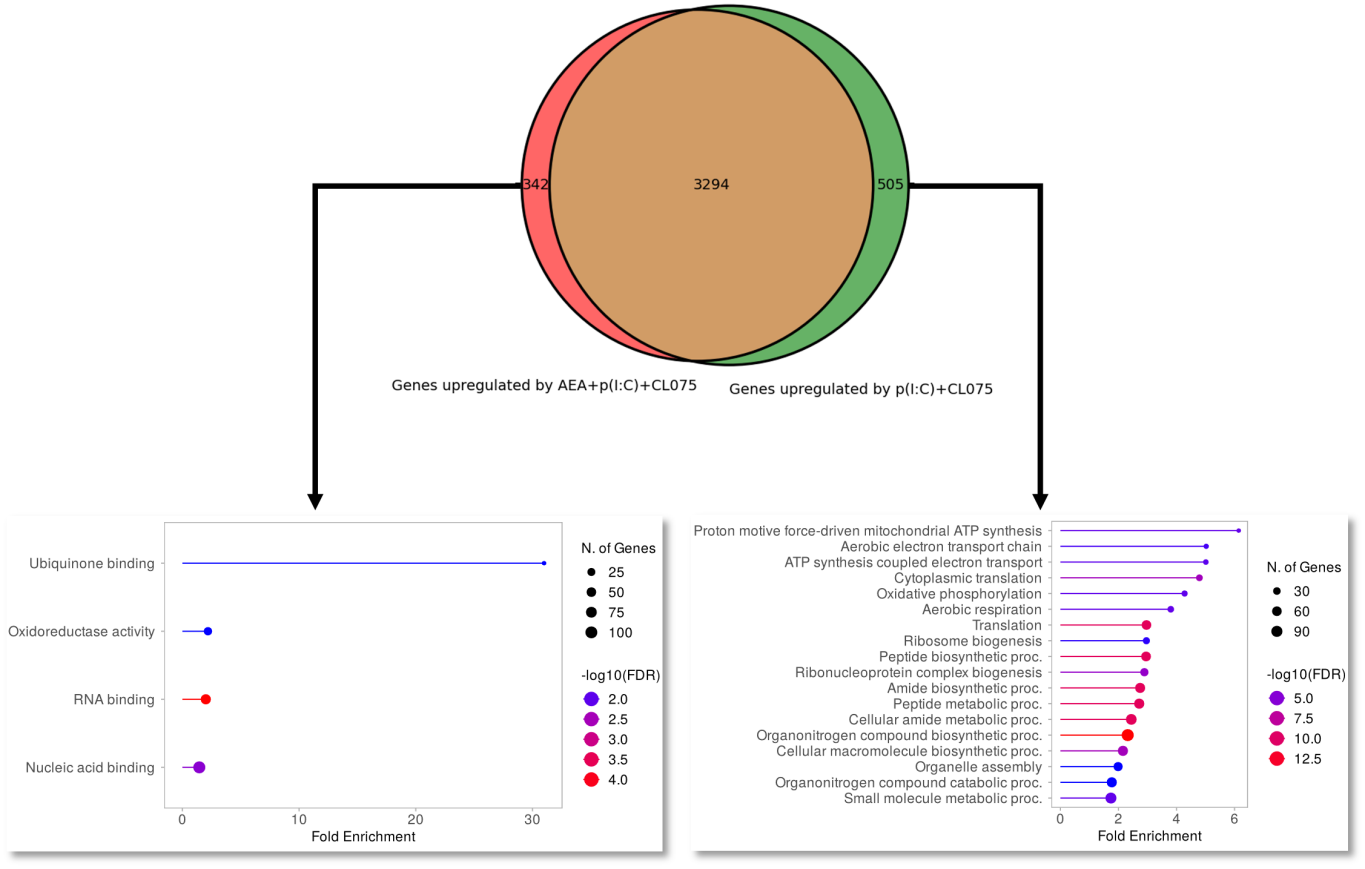
Maturation-induced switch to oxidative phosphorylation is absent from anandamide-treated moLCs. Monocytes were cultured in the presence of GM-CSF, TNFα, TGFβ and IL-4 (for 48 hrs) for 5 days to generate moLCs in the presence of 10 µM AEA, vehicle (0.1 v/v% absolute ethanol). Maturation was induced on day 4 by p(I:C) (20 µg/ml) and CL075 (0.5 µg/ml) both applied for 24 hours. Venn diagram showing number of genes upregulated by p(I:C), CL075 and AEA (left side) and p(I:C) and CL075 (right side) when compared to vehicle-treated control cells. Arrows point to upregulated pathways as determined by Gene Ontology analysis of genes that only appear in p(I:C), CL075 and AEA treated cells (marked in red on the Venn diagram, left side) or in p(I:C) and CL075 treated cells (marked in green on the Venn diagram, left side). The size of the circle corresponds to the number of induced genes, while the color of the line corresponds to the -log10 of the False Discovery Rate (FDR).

RNASeq analysis moLCs shows that differentiation in the presence of anandamide had minimal effects on the gene expression of the cells, especially compared to the changes elicited by TLR agonist treatment. A PCA plot shows that control and anandamide treated samples (in blue and red, respectively) cluster close together, as do samples treated with TLR agonists, whether they be control, or moLCs differentiated in the presence of anandamide (**Figure 5A**, in green and purple respectively). Volcano plots of transcribed genes support these findings, as only a modest number of genes show differential expression in the anandamide group compared to the control (**Figure 5B**), and between the TLR agonist-treated groups (**Figure 5C**). In contrast, when comparing control cells to TLR agonist treated samples we see a much higher number of differentially expressed genes (several hundred genes are differentially regulated by anandamide, compared to multiple thousands with TLR agonists, **Figures 5D, E**). Gene set enrichment analysis of upregulated and downregulated genes highlighted by limma-voom analysis shows that anandamide applied alone induces type II IFN receptor activity, while TLR agonists induced the expected inflammatory pathways, such as the Defense response to virus, Cellular response to stress pathways, among others (**Figures 5F-H**). Interestingly, when comparing the samples that received only TLR agonists to those that were differentiated with anandamide before TLR activation, we see that there are only minor differences between the induced pathways. To gain deeper insight into these differences between these similar groups, we used Venn diagrams to highlight those genes that are only expressed in either the former, or the latter group (**Figure 6**). When performing pathway analysis of these selected genes, we found that application of anandamide in conjunction with TLR agonists resulted in the loss of genes that take part in oxidative phosphorylation, among other metabolic pathways.

### 3.6 Anandamide-treated moLCs preferentially polarize naïve T cells towards a Th1 phenotype

Since our RNASeq analysis showed that anandamide can induce type II IFN signaling, we finally wished to examine the polarization of T cells induced by our moLCs.

**Figure 7:**
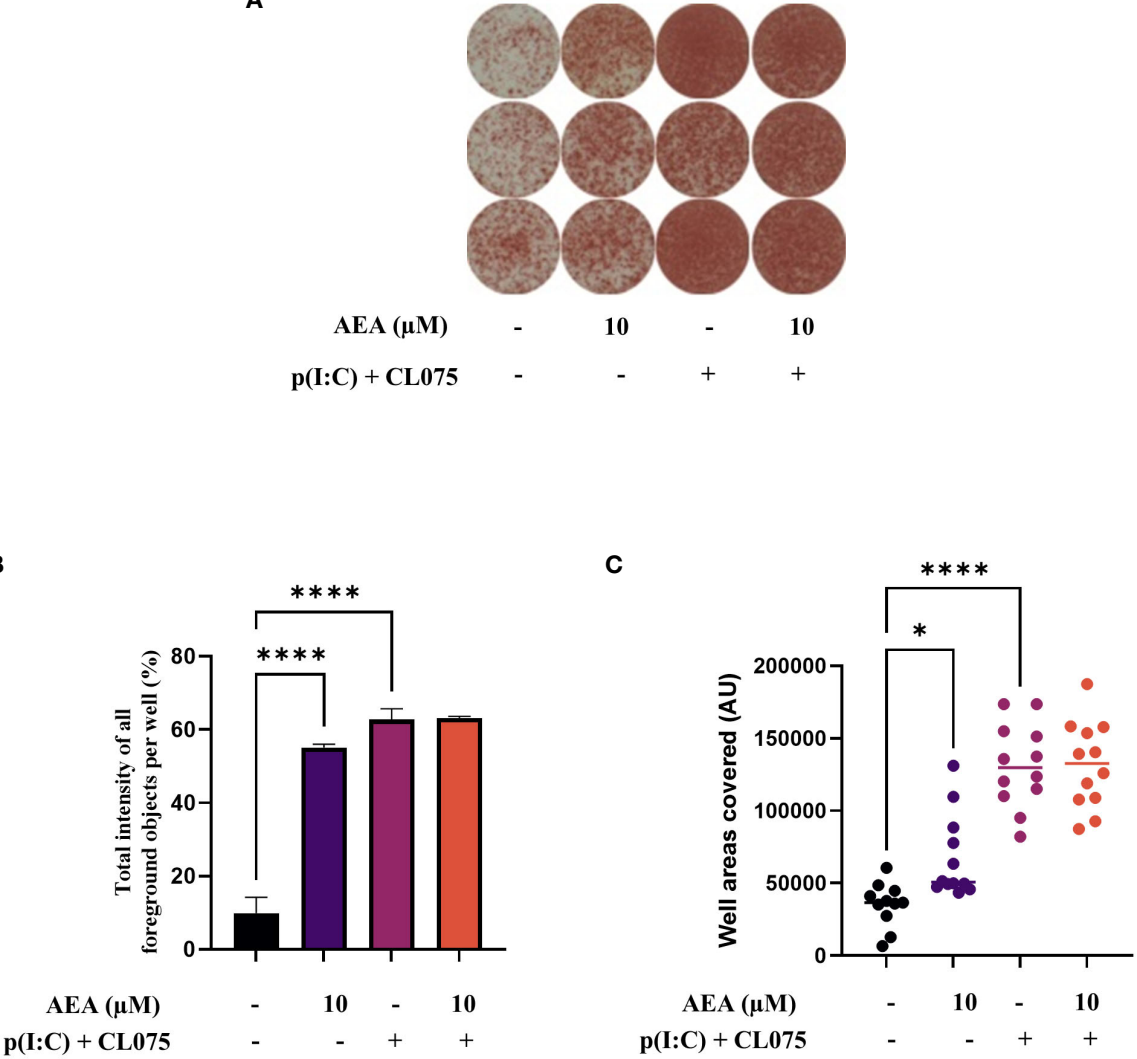
Anandamide slightly increases Th1 polarization induced by moLCs as determined by IFNγ ELISpot. Monocytes were cultured in the presence of GM-CSF, TNFα, TGFβ and IL-4 (for 48 hrs) for 5 days to generate moLCs in the presence of 10 µM AEA, vehicle (0.1 v/v% absolute ethanol). Maturation was induced by on day 4 p(I:C) (20 µg/ml) and CL075 (0.5 µg/ml), applied both for 24 hours. Representative ELISpot image showing the quantity of IFNγ producing T cells in three parallel technical repeats in brown **(A)**. The total intensity of all foreground objects per well **(B)** in panel **(A)**. The mean values and the converted well area of spot numbers were counted based on 4 independent donors **(C)**. Bar plots represent mean ± SD of representative results from N≥3 independent experiments, * p < 0.05, **** p<0.0001 compared to the marked groups as determined by repeated measures one-way ANOVA. ND, not determined. AEA, N-arachidonoylethanolamine, anandamide; CL075: TLR7/8 agonist, p(I:C), Polyinosinic:polycytidylic acid, TLR3 agonist.

Using ELISpot we were able to show that coculturing T cells with moLCs that were differentiated in the presence of anandamide induced a higher number of IFNγ positive cells compared to the control (**Figure 7A**), which could be measured by both the total intensity of all foreground objects per well, as well as the ratio of covered areas in the wells (**Figures 7B, C**, respectively). It is important to stress that in these experiments anandamide was never applied directly to T cells, only to moLCs during their differentiation.

## 4 Discussion

Endocannabinoids in general, and anandamide specifically has generally been shown to act as an endogenous brake on multiple immune cells (3, 6, 42). We started investigating the effect of anandamide on the differentiation and activation of moLCs, while ruling out the possibility of toxicity caused by the treatment. One of the significant effects of anandamide was the downregulation of CD1a on moLCs, without a concomitant change in CD207 expression (**Figures 1B, C**). As LCs in general are characterized by both CD1a and CD207 expression, the lack of change in the latter hints that anandamide does not inhibit moLC differentiation, but selectively downregulates CD1a expression. Surprisingly anandamide increased the expression of MHCII molecules, CD86 and even CCR7 (**Figures 1E-G**). This hints that moLCs differentiated in the presence of anandamide are more effective at presenting antigen to, and activating naïve T cells, and that through their increased CCR7 expression are more likely to migrate to draining lymph nodes from the skin, further supporting the relatively selective effect on CD1a as opposed to more general inhibition. We could also rule out the possibility that anandamide was simply toxic to the cells, as we saw no increase in 7-AAD positive cells (**Figure 1A**). This was an important control experiment, as others have reported significant cell death with similar concentrations in keratinocytes [10 μM, (43)], melanoma cells [13.5 μM, (44); >5 μM, (45)], sebocytes [>10 μM, (46)], and murine DCs [>5 μM, (47)].

Further supporting the idea that anandamide in the context of moLCs is not purely anti-inflammatory we found that activation of the cells with TLR3 and TLR7/8 agonists was just as effective in cells differentiated in the presence of the endocannabinoid as in control cells (**Figures 1B-F**). Interestingly, the TLR agonists did not increase the expression of CCR7 in control cells, which corresponds to the “activated LC” phenotype established by Liu et al., which is characterized as CCR7^low^ as opposed to the “migratory LC” that have high CCR7 expression (26).

In contrast to anandamide, 2-AG had no significant effects on the markers typically investigated on antigen presenting cells such as LCs and DCs (34), so we excluded this treatment from subsequent experiments (**Supplementary Figure 1**). This lack of effect from 2-AG was not completely unexpected, since although it was able to induce maturation of the DC2.4 DCs line, it did not influence either CD4^+^ or CD8^+^ T cells in the tumor microenvironment in mice (48).

CD1a is primarily expressed on the surface of cortical thymocytes, LCs in the skin and mucosal surfaces, and some DCs. The CD1a molecule has a structure similar to major histocompatibility complex (MHC) molecules, which are involved in presenting peptide antigens to T cells. CD1 molecules are specialized for presenting lipid antigens rather than peptide antigens. There are multiple possible mechanisms that can explain the decrease in CD1a, as this might occur due to increased cAMP concentration inside the cell during its differentiation (49), binding of anandamide as a lipophilic molecule to CD1a which can influence its trafficking and result in lower expression overall. In monocyte derived DC (moDC) cultures the presence of lipoproteins in culturing media causes an activation of peroxisome proliferator-activated receptor gamma, which decreases the expression of CD1a (50). This drop in CD1a could conceivably result in a decreased capability to activate naïve T cells, as there are certain T cell populations that are restricted to CD1a (51), however in our experiments we found the exact opposite effect: anandamide was capable of significantly increasing the ratio of proliferating allogenic T cells (**Figure 4**). As CD1a-restricted T cells have been implicated in multiple inflammatory skin diseases such as psoriasis, atopic dermatitis and even contact hypersensitivity, this targeted decrease of CD1a expression might be beneficial in these diseases (52).

After investigating the effect of anandamide on the differentiation and activation of the cells, we next evaluated the function of moLCs differentiated in the presence of anandamide. Even though anandamide showed some proinflammatory effects via the markers detailed above, when investigating its effects on cytokine secretion it appeared to induce anti-inflammatory results. At high concentration (10 μM) anandamide decreased the TLR3 and TLR7/8 induced production of CXCL8, IL-6, IL-10, IL-12 (**Figures 2A-D**). Similar effects have been described by others, for example in J774 macrophages, anandamide hinders LPS-induced NO and IL-6 synthesis in a concentration-dependent manner (17). Anandamide also effectively modulates the activity of macrophages and monocytes, displaying a regulatory impact contingent on both dosage and time. Previous results on human peripheral blood mononuclear cells (PBMCs) have shown anandamide diminishes the generation of IL-6 and CXCL8 at lower concentrations, while completely suppressing the release of TNFα, IFNγ, and IL-4 at higher concentrations (15). Further in line with our findings, anandamide also suppresses TLR7/8-induced IL-12 and IL-6 production in human myeloid DCs and inhibits IFNα production in human plasmacytoid DC (53). Furthermore, anandamide also inhibits the proliferation and secretion of cytokines, such as IL-2, TNFα, and IFNγ, from activated human peripheral T lymphocytes (15, 16). These results show that our observation that anandamide dampens cytokine secretion is not unique to moLCs.

Anandamide has been shown to act on a wide range of cellular receptors, including the non-selective cation channel TRPV1 (54), the activation of which can lead to increases in intracellular calcium. As calcium signaling is an important mediator in cytokine secretion, maturation, and a host of other immune functions in both murine and human cells (55–59) we next wished to investigate the effect of anandamide on the intracellular calcium concentration of moLCs. Although anandamide increased the intracellular calcium concentration of moLCs as determined by fluorescent calcium imaging, not all cells were equally responsive to anandamide (**Figure 3**, **Supplementary Videos 1**-**4**). In fact, the calcium transient that can be observed occurs relatively late after the application of anandamide (with an approximate maximal response at 150 seconds), which stands in contrast to the response elicited by capsaicin on a similar moLC model (33). According to the kinetics of the calcium response, and the low number of responsive cells, it is unlikely that the effects of anandamide are dependent on TRPV1 activation on our moLCs. moLCs also express other TRP channels, such as TRPV2 and TRPV4 (34), but anandamide has not been described to act on these channels.

Based on the above presented results anandamide seems to induce partly antagonistic effects: it boosts antigen presentation and subsequently T cell proliferation, both of which could be considered proinflammatory responses, while on the other hand, cytokine and chemokine production is decreased by anandamide treatment. To gain deeper insight into the induced responses we performed RNASeq analysis of moLCs differentiated in the presence of anandamide, with and without activation by TLR ligands (**Figures 5**, **6**). This analysis highlighted two important details regarding the transcriptome of moLCs. First, we found that anandamide treatment upregulated type II IFN receptor activity, which suggests that it might help polarize T cells activated by moLCs towards a Th1 phenotype. Secondly, we found that in cells activated with TLR agonists there is an enrichment of genes involved in oxidative phosphorylation (**Figure 6**, right panel), which is absent from cells differentiated in the presence of anandamide. This is accompanied by enrichment in other metabolic pathways, such as aerobic electron transport chain, ATP synthesis coupled electron transport, aerobic respiration, and others. Typically, activated myeloid cells tend to be highly glycolytic with little or no flux through oxidative phosphorylation. Activation of DCs, which share many functional characteristics with LCs, results in a switch to glycolysis to support *de novo* lipid biosynthesis, facilitating the expansion of the endoplasmic reticulum and Golgi apparatus and increasing the biosynthetic capacity of the cells (60). The concomitant decrease in oxidative phosphorylation typically results in a decrease in activated DC lifespan, which can be rescued by continued availability of oxidative phosphorylation to the cells, by for example mTOR inhibition (61). As we saw no decrease in either the viability or the T cell activating capability of anandamide and TLR agonist treated moLCs compared to moLCs only treated with p(I:C) and CL075, this metabolic shift is most likely insufficient in scope to decrease the longevity of the cells. Another consequence of this shift could be the accumulation of metabolic intermediaries required for the *de novo* synthesis of cytokines, as seen in DCs (62). As anandamide seems to interfere with this shift, this might underlie the decrease in cytokine production we observed (**Figure 2**). It is also important to note that increased oxidative phosphorylation is a hallmark of tissue resident macrophages, which are ontogenically close relatives of LCs. Upregulation of this pathway in activated LCs would increase their longevity, which could be beneficial in the case of cells that remain in the skin (which is supported by their low CCR7 expression), but would likely not be required for migratory LCs with high CCR7 expression (63). It is important to note that these effects on the metabolism of moLCs should be investigated in more detail, as changes in the transcriptome do not necessarily translate to concomitant changes in the proteome. Further, targeted experiments are required to substantiate these preliminary findings in subsequent investigations. The effect of anandamide on cellular metabolism is not completely surprising, however. A recent manuscript (64) details a novel method of identifying cellular targets of cannabinoids, and they identify 64 new targets, many of which are associated with the mitochondria. Of these 64 novel targets 10 are directly involved in aerobic respiration pathways, which might explain our own gene set enrichment results.

In contrast to the negative effect of anandamide on chemokine/cytokine secretion, we found that moLCs treated with anandamide alone stimulated the proliferation of naïve T cells, albeit not to the level reached with combined TLR agonists (**Figure 4**). The combination of anandamide and TLR agonists did not exhibit a notably additive effect; however, a slight increase was observed between the p(I:C) + CL075 group and the anandamide + p(I:C) + CL075 group (**Figure 4A**, with representative histogram plots depicted in **Figure 4B**).

Although most literature data are from mouse models, these show that LCs are able to induce Th1, Th2 and Th17 (65, 66) and most likely, regulatory T cells (67). When the immune response of moDC and moLC were investigated upon stimulation with TLR agonists, with or without proinflammatory cytokines TNFα and IL-1β minor differences could be observed in T cell polarization. Bacterial agonists selectively up-regulated CD83 and CD86 expression and induced Th1 and Th17 response in moDC, which showed higher migratory capacity compared to moLCs. MoLC activation with LPS enhanced the mature state (CD83, CD86 marker expression), and also increased IL-12p70, IL-23, and IL-6 production, and induced Th1 cytokine production by CD4^+^ T cells (68). In accordance with these findings, in our experiments, p(I:C) and CL075 stimulation up-regulated the costimulatory molecules and increased IL-12 secretion in moLCs (**Figure 2D**).

As RNASeq analysis revealed an increase in IFN gamma receptor activity in anandamide treated moLCs (**Figure 5F**), as well as a marked increase in IL-12 production in TLR agonist treated moLCs (**Figure 2D**), we next wished to investigate the Th1 polarization of activated T cells. Using ELISpot we could determine that anandamide alone can increase both the intensity of staining and the area covered by IFNγ positive T cells, indicating that there is an increase in proliferating T cells (supporting **Figure 4**), as well as their IFNγ content. The combined application of p(I:C) and CL075, as expected, resulted in a much stronger response, which was not influenced further by the addition of anandamide during the differentiation of moLCs. It is important to note that in these experiments T cells were not treated with anandamide directly, as this is known to decrease both TNFα and IFNγ production from primary human T lymphocytes at low micromolar concentrations, and can induce apoptosis at high micromolar concentrations (16, 42), but rather the endocannabinoid was only applied to moLCs during their differentiation.

Based on the results presented above we can state that moLCs join the ranks of immune cells under the regulation of the ECS. Although ECs in general, and anandamide especially are often considered anti-inflammatory, the nuanced effects on maturation, cytokine secretion, T cell proliferation and polarization presented above underline the fact that an integrated overview of cell-specific effects is required to accurately predict the role of the ECS in regulating immune responses.

As our knowledge of the ECS continues to expand, it is becoming increasingly clear that this intricate network plays a pivotal role in maintaining human health and offers promising avenues for therapeutic interventions. As CD1a signaling is implicated in common inflammatory skin disorders, the mitigating effect of anandamide on CD1a expression holds promise to selectively dampen inflammation induced by CD1a restricted T cells, without impacting systemic immune responses as antigen presentation through the MHC pathway is not impacted in a negative way. The evolving understanding of the ECS’s involvement in skin health opens up exciting opportunities for developing novel cannabinoid-based therapies for dermatological conditions and promoting overall skin well-being.

## Data availability statement

The datasets generated for this study can be found in the Gene Expression Omnibus repository hosted at the National Library of Medicine, under accession number GSE266075. https://www.ncbi.nlm.nih.gov/geo/query/acc.cgi?acc=GSE266075.

## Ethics statement

The studies involving humans were approved by Regional and Institutional Ethics Committee of the University of Debrecen’s Faculty of Medicine (Debrecen, Hungary; approval number: OVSZK 3572-2/2015/5200). The studies were conducted in accordance with the local legislation and institutional requirements. The human samples used in this study were acquired from a by- product of routine care or industry. Written informed consent for participation was not required from the participants or the participants’ legal guardians/next of kin in accordance with the national legislation and institutional requirements.

## Author contributions

ZP: Conceptualization, Formal Analysis, Investigation, Methodology, Visualization, Writing – original draft, Writing – review & editing. DH: Writing – review & editing. PM: Writing – review & editing. TF: Writing – review & editing. KP: Writing – original draft, Writing – review & editing. AB: Resources, Writing – review & editing. AS: Conceptualization, Formal Analysis, Funding acquisition, Project administration, Supervision, Writing – original draft, Writing – review & editing.
