# Supplementary material for: Anandamide modulation of monocyte-derived Langerhans cells: implications for immune homeostasis and skin inflammation
Source: Front Immunol. 2024 Jun 24;15:1423776. doi: 10.3389/fimmu.2024.1423776 (PMC11228147; doi:10.3389/fimmu.2024.1423776)
Supplement: Supplementary file 5 [file DataSheet_1.docx]

Supplementary Material

# Supplementary Data

##
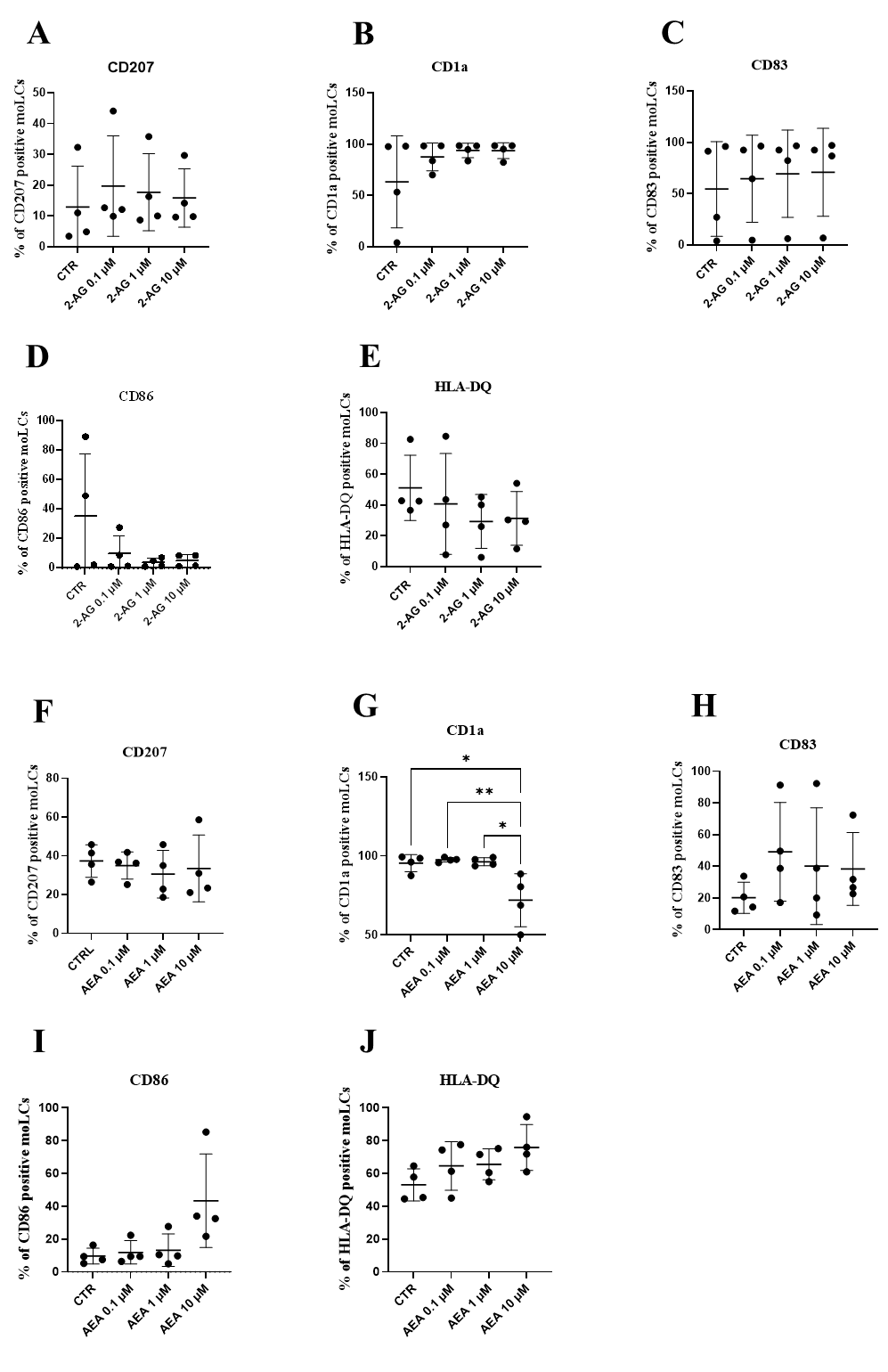
Supplementary Figures

**Supplementary Figure 1.** Broad concentration spectrum (0.1 - 10 µM) effects of anandamide and 2-AG on differentiation and maturation markers

Monocytes were cultured in the presence of GM-CSF, TNFα, TGFβ and IL-4 (for 48 hrs) for 5 days to generate moLCs in the presence of 0.1, 1, 10 µM 2-AG, 0.1, 1, 10 µM AEA, vehicle (0.1 v/v% absolute ethanol). N=4, lines mark mean±SD. Percentage of cells positive for CD207 (**A, F**), CD1a (**B, G**), CD83 (**C**, **H**), CD86 (**D, I**), HLA-DQ (**E, J**), following the indicated treatments.

*p < 0.05, ** p < 0.01, as indicated (determined by repeated measures one-way ANOVA). Individual donors are represented by symbols. CTRL: moLCs treated with 0.1 v/v% absolute ethanol, AEA: N-arachidonoylethanolamine, anandamide, 2-AG: 2-Arachidonoylglycerol


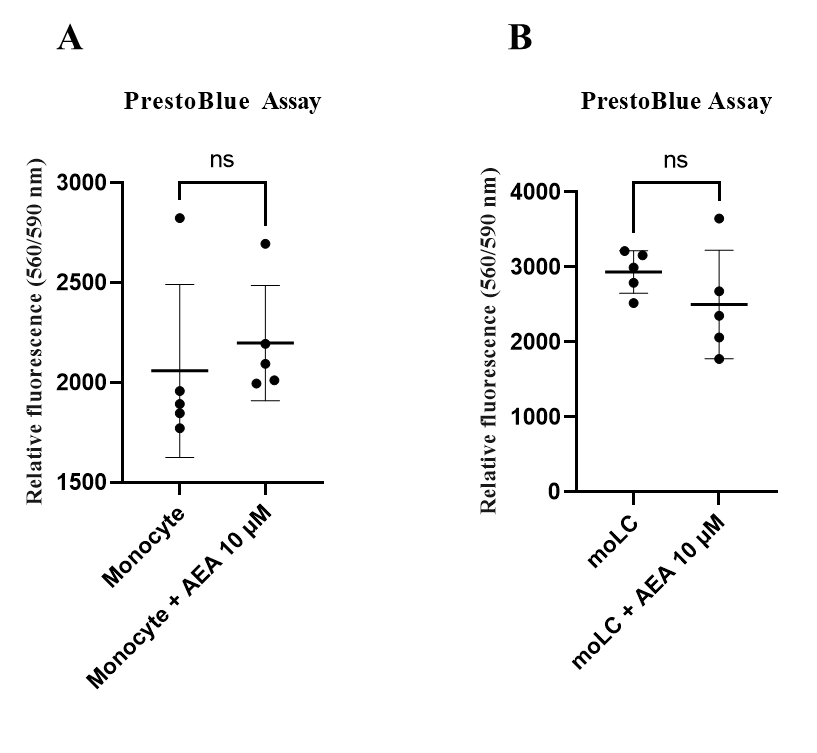


**Supplementary Figure 2.** Anandamide do not decrease viability of monocytes and moLCs.

Monocytes were cultured for 1 day without any added cytokines (monocytes), or for 5 days in the presence of GM-CSF, TNFα, TGFβ and IL-4 (for 48 hrs) to generate moLCs in the presence of 10 µM AEA, vehicle (0.1 v/v% absolute ethanol). N=5, lines mark mean±SD. Determination of monocytes viability (**A**) and moLCs viability by PrestoBlue Assay (**B**). Individual donors are represented by symbols. Monocytes and moLCs treated with 0.1 v/v% absolute ethanol, AEA: N-arachidonoylethanolamine, anandamide.

**Supplementary Figure 3.** Heatmap of chemokine receptors expressed on moLCs.

Monocytes were cultured in the presence of GM-CSF, TNFα, TGFβ and IL-4 (for 48 hrs) for 5 days to generate moLCs in the presence of 10 µM AEA, vehicle (0.1 v/v% absolute ethanol). Maturation was induced on day 4 by p(I:C) (20 µg/ml) and CL075 (0.5 µg/ml), both applied for 24 hours. N≥3 mean±SD. Heatmap shows expression of chemokine receptors. Monocytes and moLCs treated with 0.1 v/v% absolute ethanol, AEA: N-arachidonoylethanolamine, anandamide. AEA: N-arachidonoylethanolamine, anandamide, CL075: TLR7/8 agonist, p(I:C): Polyinosinic:polycytidylic acid, TLR3 agonist
